# The thrombopoietin receptor: revisiting the master regulator of platelet production

**DOI:** 10.1080/09537104.2021.1925102

**Published:** 2021-06-07

**Authors:** Ian S. Hitchcock, Maximillian Hafer, Veena Sangkhae, Julie A. Tucker

**Affiliations:** 1 York Biomedical Research Institute, Department of Biology, University of York, York, UK; 2 Department of Biology and Center of Cellular Nanoanalytics, University of Osnabrück, Osnabrück, Germany; 3 Center for Iron Disorders, Department of Medicine, David Geffen School of Medicine at UCLA, Los Angeles, California, USA

**Keywords:** Thrombopoietin, thrombopoietin receptor, platelets, megakaryocytes, signaling

## Abstract

Thrombopoietin (TPO) and its receptor, MPL, are the primary regulators of platelet production and critical for hematopoietic stem cell (HSC) maintenance. Since TPO was first cloned in 1994, the physiological and pathological roles of TPO and MPL have been well characterized, culminating in the first MPL agonists being approved for the treatment of chronic immune thrombocytopenia in 2008. Dysregulation of the TPO-MPL signaling axis contributes to the pathogenesis of hematological disorders: decreased expression or function results in severe thrombocytopenia progressing to bone marrow failure, while hyperactivation of MPL signaling, either by mutations in the receptor or associated Janus kinase 2 (JAK2), results in pathological myeloproliferation. Despite its importance, it was only recently that the long-running debate over the mechanism by which TPO binding activates MPL has been resolved. This review will cover key aspects of TPO and MPL structure and function and their importance in receptor activation, discuss how these are altered in hematological disorders and consider how a greater understanding could lead to the development of better-targeted and more efficacious therapies.

## Introduction

Thrombopoietin (TPO) was first described in the late 1950s as a humoral factor induced in response to thrombocytopenia and capable of increasing circulating platelet count[1]. Despite many attempts to isolate and clone TPO, the hormone remained elusive until 1986, when the seemingly unrelated discovery of the myeloproliferative leukemia virus (v-MPL), a retrovirus that causes acute leukemia and polycythemia in mice[2], provided a clear path for the isolation and characterization of TPO several years later. Subsequent analysis of v-MPL and its cellular homologue, MPL, revealed it to be a member of the type I cytokine receptor family and the likely receptor for TPO [3,4]. Aided by the cloning of MPL, several groups successfully cloned and isolated TPO in 1994 [5–8].

Research over the following two decades revealed TPO-MPL as a key hematopoietic cytokine signaling complex, not only for the regulation of megakaryocyte differentiation and platelet homeostasis, but also for hematopoietic stem cell (HSC) self-renewal and proliferation. The therapeutic potential of TPO was realized in 2008 with the clinical approval of two MPL agonists, romiplostim[9] and eltrombopag[10] for the treatment of chronic immune thrombocytopenia (ITP) and later for aplastic anemia.

Despite their role in biology and medicine, the mechanisms of TPO-MPL interaction and activation are not wholly understood. Defining these interactions and how they are altered in hematological disorders, such as the myeloproliferative neoplasms and thrombocytopenias, could aid the identification and development of novel therapeutic agents. Indeed, recent evidence highlights the remarkable complexity of receptor activation in both physiological and pathological contexts [11–13]. These findings may in turn generate new possibilities for fine-tuning signaling outputs from MPL, allowing greater and more precise control of HSC maintenance and megakaryocyte differentiation.

## Thrombopoietin

**Figure 1.**
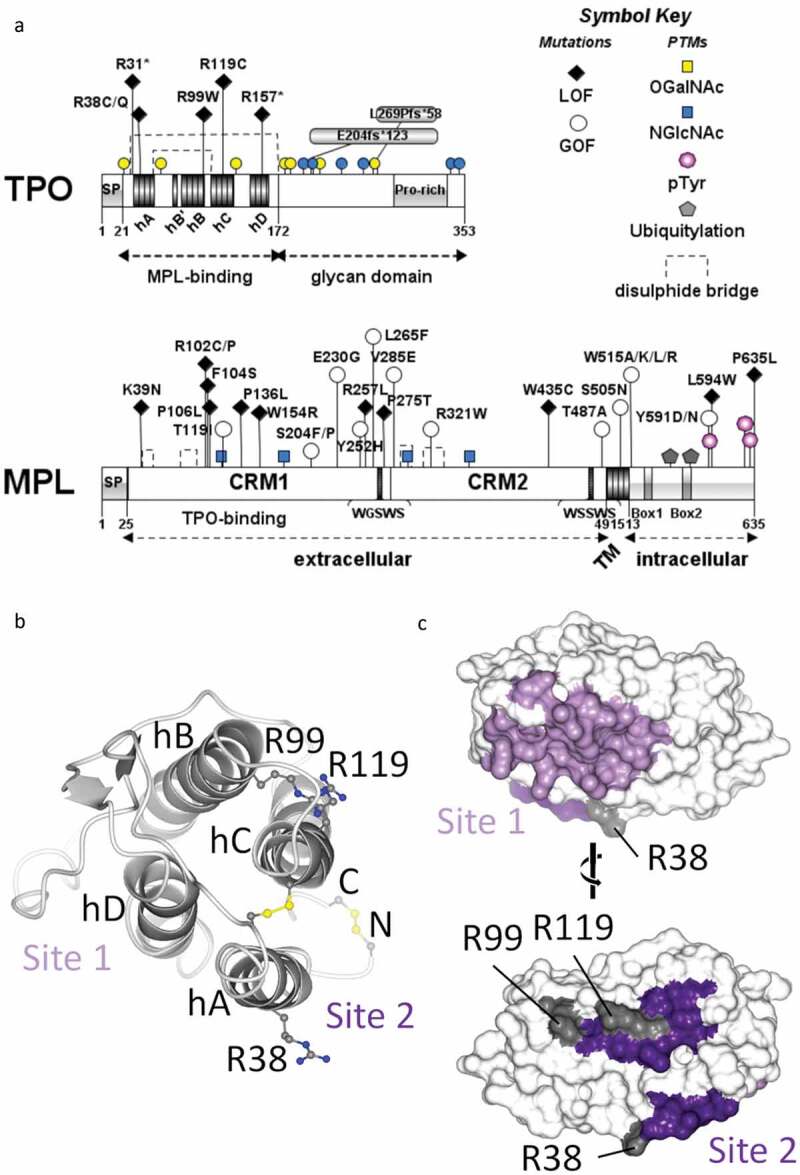
**Schematic representations of TPO and MPL proteins**. (**A**) Domain diagrams showing selected gain-of-function (GOF) and loss-of-function (LOF) mutations, conserved sequence motifs and post-translational modifications. The helices of the MPL-binding domain within TPO are labeled hA through hD. SP (signal peptide), CRM (cytokine receptor module), TM (transmembrane domain). (**B**) Ribbon representation of the 4-helical bundle MPL-binding domain of human TPO with the N- and C-termini facing away from the viewer, such that the two MPL-binding sites are shown to the left and right respectively. Helices are labeled as in **A**. The four conserved cysteines and three arginines mutated in hereditary thrombocytopenia are labeled and drawn in ball-and-stick representation (carbon atoms in dark gray, nitrogen in blue, sulfur in yellow). (**C**) Molecular surface of the MPL-binding domain of TPO colored according to predicted high (site 1) and low (site 2) affinity binding sites for MPL[14]. The MPL-binding sites are highly conserved and lie on opposing faces of TPO. Residues mutated in hereditary thrombocytopenia are indicated. Views are rotated 180º with respect to one another. Panel A prepared using Illustrator for Biological Sequences[15]; panels B-C prepared using chain V from PDB 1V7M[14], and CCP4MG[16]. Residues are numbered according to the human TPO and MPL precursor proteins with Uniprot IDs P40225 and P40238, respectively

**Figure 2.**
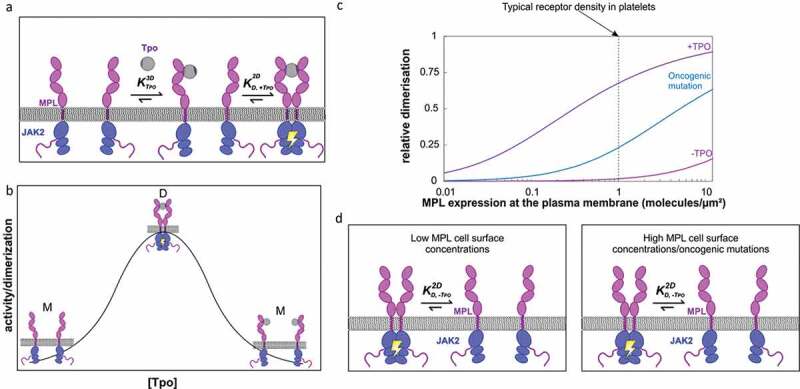
**MPL receptor dimerization characteristics**. Two-step assembly mechanism (**A**) for the formation of active homodimeric signaling complexes accounts for the (**B**) bell-shaped TPO dose-response curve. At physiological receptor densities and in the absence of TPO (gray), MPL (pink) and the associated intracellular JAK2 (blue) are monomeric and inactive. At low concentrations of TPO (below K_1_ or K^3D^_D_), most MPL at the cell surface are not bound to TPO (and are thus monomeric (M) and inactive), whilst a small number are bound to TPO in a binary (1:1), non-signaling competent complex. As the concentration of TPO rises, the proportion of binary complexes on the cell surface increases, and there is an increased likelihood that these will encounter a second MPL monomer and form a ternary (2:1 MPL:TPO), active complex (D) with the 2-dimensional binding affinity K_2_ (or K^2D^_D_). Ternary complex formation and downstream signaling (indicated by yellow ‘lightning’) reach a maximum when the concentration of TPO equals K_1_. Finally, as the concentration of TPO exceeds K_1_, MPL monomers become saturated with TPO and binary non-signaling complexes (M) predominate. The two different MPL-binding sites on TPO are colored as in Figure 1, in light and dark purple, respectively. Panel **B** is a theoretical representation of the bell-shaped dose-response curve. (**C, D**) Enhanced receptor dimer stability, as a result of oncogenic mutations in MPL and/or JAK2 leads to the TPO-independent signaling that underlies thrombocytic disease. Increased receptor density also leads to MPL dimer formation in the absence of cytokine. Panel **C** prepared using data from[11]

The human *THPO* gene encodes a secreted protein composed of a signal peptide followed by two functional domains; an N-terminal MPL-binding domain and a C-terminal, so-called glycan domain (Figure 1a). The crystal structure of the N-terminal domain of TPO reveals a classical 4-helix bundle, similar to erythropoietin (EPO) and growth hormone (GH), held together by two conserved disulfide bridges[14] (Figure 1b). In common with EPO and GH, TPO has two non-identical binding sites for its receptor; a high-affinity site (site 1) with binding affinity K_1_ in the nanomolar range, and a lower affinity site (site 2) with binding affinity K_2_ in the micromolar range[14] (Figure 1(b-c)). These are located on opposite sides of the 4-helical bundle, such that the active signaling complex comprises one molecule of TPO bound to two molecules of MPL (Figure 2(a-b)). It is important to note that the binding affinities reported by Feese and colleagues[14] were measured in solution, using recombinant, soluble, monomeric receptor ectodomain and the MPL-binding domain of TPO, and thus may not accurately reflect the situation in cells. In particular, the three-dimensional K2 measured in solution may underestimate the true binding affinity on cells, which is a two-dimensional binding event (see below and Figure 2a). Characterization of the impact of homology model-based site-directed mutants of TPO on MPL binding and cellular proliferation, as well as biophysical characterization of TPO and its competing interactions with soluble MPL and neutralizing antibodies, confirmed the two-step assembly model, in which site 1 mediates the binding of the first MPL monomer, whilst site 2 is required for interaction with a second monomer to form a ternary complex that is competent for downstream signaling. Mutations in the predicted site 1 reduced affinity for MPL in a phage ELISA, whilst those in the predicted site 2 were permissive for MPL binding, but showed reduced specific activity in proliferation assays [14,17–20]. Similarly, the epitope for neutralizing antibodies that blocked both initial binding of TPO to MPL and bioactivity mapped to the predicted site 1, whilst neutralizing antibodies that did not prevent receptor association bound to an epitope in the predicted site 2 (see below)[17]. Interestingly, levels of TPO in plasma and serum from healthy individuals [21–23] are more than 1000-fold below the values of K1 measured *in vitro*[14] and cells[11]. This suggests that under normal physiological conditions, the majority of receptors on the cell surface will not be bound to TPO. Of note, the reported values of TPO in serum and plasma may not be representative of the levels of TPO in the bone marrow milieu nor of the local concentration of TPO at the cell surface.

In contrast to EPO and GH, TPO possesses an additional, predicted intrinsically disordered C-terminal domain, which is heavily glycosylated (six *N*-linked and four *O*-linked sites)[24]. The glycan domain plays a role in correct trafficking of the cytokine through the secretory system [25,26].

**Figure 3.**
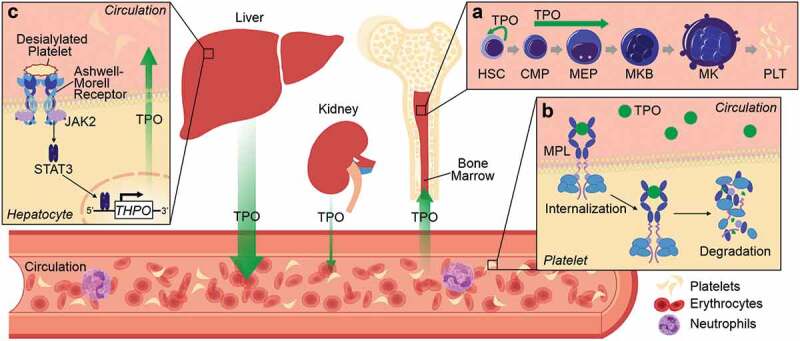
**Expression, function and regulation of TPO**. TPO is mainly produced in the liver and to a lesser extent in the kidneys. Once released into the circulation TPO drives megakaryocyte differentiation in the bone marrow, increasing the numbers of circulating platelets (**A**). There are currently two proposed mechanisms of TPO regulation. The “platelet sponging theory” (**B**) postulates that MPL on platelets bind circulating TPO, inducing internalization and degradation of the complex, thereby allowing circulating platelets to directly influence megakaryopoiesis and production of new platelets. A more recent model (**C**) suggests that changes in surface glycan composition in aged or senescent platelets promotes their removal; desialylated platelets interact with Ashwell-Morrell receptors on hepatocytes, activating Janus kinase (JAK)/signal transducer and activator of transcription (STAT) signaling pathways to increase *THPO* transcription. HSC (hematopoietic stem cell), CMP (common myeloid progenitor), MEP (megakaryocyte erythrocyte progenitor), MKB (megakaryoblast), MK (megakaryocyte), PLT (platelet)

TPO is primarily expressed in the liver [27,28] and levels of circulating TPO are controlled by multiple regulatory mechanisms, from transcription (promoter usage and alternative splicing) and translation[29] through secretion and ultimately clearance. Physiological levels of plasma TPO are usually inversely proportional to platelet count, although sensing mechanisms regulating TPO production and release by the liver remain controversial [30–32]. It is likely, however, that a combination of these mechanisms (detailed in Figure 3) is responsible for the regulation of plasma TPO levels.

## Thrombopoietin Receptor - MPL

Along with the EPO, GH and prolactin (PRL) receptors, MPL (also called CD110 or TPOR) belongs to the group 1 cytokine receptor sub-family, and comprises an extracellular cytokine-binding domain linked via a transmembrane spanning helix to a cytosolic domain that serves as a binding site for intracellular signaling proteins [33,34] (Figure 1a). Critically, these receptors lack intrinsic kinase activity, instead relying on interactions with JAK proteins to initiate intracellular signaling. In the case of MPL, signaling is predominantly driven through JAK2^34^.

In contrast to EPOR, GHR and PRLR, no experimental structures are available for MPL in the public domain, although an all-atom molecular dynamics simulation of the membrane-embedded MPL transmembrane and intracellular domains (TM-ICD) in complex with JAK2 was published [11]. The MPL extracellular domain (ECD) differs from EPOR in that it comprises two, rather than one, cytokine receptor modules. Each cytokine receptor module (CRM) consists of two subdomains, the first of which adopts an immunoglobulin (Ig)-like fold, and contains four conserved cysteine residues which form two disulfide bridges, whilst the second resembles a fibronectin type III (FnIII) domain, and contains a WSXWS motif, characteristic of the cytokine receptor family [33,35]. By analogy with EPOR, along with mutational studies, the TPO-binding site is predicted to be primarily located in the first cytokine receptor module (CRM1) at the intersection between subdomains [36,37]. Sequence homology suggests that the overall structures of both CRM1 and CRM2 will resemble that of the EPOR ECD; however, the relative orientations of CRM1 and CRM2 with respect to one another and the plasma membrane remain unclear.

The MPL ECD is connected to the intracellular domain (ICD) via a single-pass transmembrane (TM) helix flanked by juxtamembrane (JM) regions. Spectroscopic studies of monomeric JM-TM peptides in lipid bilayers are consistent with a ‘split’ helical structure in which the extracellular JM sequence forms a short helix lying parallel to the plane of the membrane, whilst the TM helix is tilted with respect to the membrane normal [38,39]. Upon dimerization, the ‘split’ helices appeared to coalesce and their tilt angle reduced[39]. These and earlier studies identified a binding site for the MPL agonist, eltrombopag, within the TM helical region, most likely in the vicinity of H499^10^ [38–40].

The MPL ICD is predicted to be largely intrinsically disordered in the absence of binding partners[41]. Tyrosines in the ICD are phosphorylated upon receptor activation (Figure 1a) and recruit signal transduction proteins to the membrane and/or within proximity of JAK2. JAK2 associates with MPL via binding of the JAK2 FERM-SH2 domain to the MPL Box 1 and 2 sequences (Figure 1a), which were mapped via mutational studies[42]. The binding affinity of the ICD peptide for JAK2 in solution has yet to be determined, and the stability of this interaction appears to be critically dependent on the presence of the plasma membrane. In addition to interacting with MPL, the JAK2 FERM-SH2 domain anchors the receptor/kinase complex to the plasma membrane via electrostatic and hydrophobic interactions[11]. Three tyrosines (Y591, Y626 and Y631) located at the distal end of the ICD are rapidly phosphorylated in response to TPO stimulation [43,44]. Y626 is the primary phosphorylation site, whereas Y631 is a secondary site; both sites are required for maximal TPO-mediated proliferation and activation of downstream signal transduction [44–46]. Conversely, phosphorylation of Y591 appears to down-regulate MPL activity by limiting receptor activation[43] and driving receptor internalization[47].

MPL is expressed by HSCs from the earliest stage of development[48] and is critical for megakaryocyte development[50]. Decreased receptor expression or function can cause severe thrombocytopenia in humans[49], a phenotype recapitulated by *Mpl-null* mice, which have only ~5-10% the number of megakaryocytes and circulating platelets compared to *wild-type* animals[50]. Evidence suggests the primary role of TPO-MPL is to promote megakaryocyte lineage selection in myeloid progenitor cells, rather than differentiation of more mature megakaryocytes. In mice, ablation of *Mpl* specifically in the megakaryocyte lineage (*Mpl-floxed/Pf4cre*) not only failed to reduce platelet count, but actually drove myeloproliferation, presumably as a consequence of ineffective TPO clearance due to absence of MPL on platelets[51]. Interestingly, there is also some evidence that TPO may prime platelets for activation. Increased levels of plasma TPO in patients with unstable angina appears to increase monocyte-platelet aggregates and enhances platelet aggregation in response to certain agonists [52,53]. Fortunately, however, there is no evidence of an increased risk of thrombosis in patients receiving TPO agonists [54,55].

## Mechanisms of MPL Activation by TPO

There is some debate in the literature as to whether TPO drives activation of MPL via receptor dimerization or by inducing conformational changes in a pre-formed receptor dimer. In this section, we will try to consolidate previously published work to suggest a feasible model of receptor activation.

Early work provided two lines of evidence in support of TPO-mediated MPL dimer formation driving signaling; cysteine mutations at a proposed dimer interface resulted in constitutive activation[56], whilst the Fab fragments of agonist monoclonal[57], and polyclonal antibodies[58] could not support cell proliferation.

Since 1998, it has been generally accepted that MPL and other group I cytokine receptors exist as pre-formed dimers in the plasma membrane [40,59–64] and that TPO binding induces a conformational change in the MPL dimer, which is then propagated by some mechanism across the plasma membrane to result in activation of the receptor-associated JAK2. The two non-identical MPL-binding sites, which are located on opposing faces of TPO (Figure 1(b-c)), are predicted to bind spatially similar surfaces on two separate molecules of MPL. The predicted TPO-binding site on MPL is located at the interface between the Ig-like and FnIII domains of CRM1. The ternary complex itself is, therefore, almost, but not perfectly symmetric. Local side-chain re-organization both within TPO and the MPL ECD is likely required to accommodate TPO binding and receptor dimerization, in a similar manner to that observed for GH binding to GHR[33]. These conformational changes could contribute to signaling across the plasma membrane; however, given the predicted flexibility of the intervening protein domains, such a mechanism seems unlikely. On the other hand, studies using artificially dimerized, chimeric MPL fusion proteins, in which the receptor ECD was replaced with a dimeric coiled coil derived from the *S. cerevisiae* transcriptional activator, Put3^64^, have demonstrated that different orientations of the TM within the dimer display differences in signaling outcome. These differences in orientation are then transmitted to the ICD-associated JAK2, presumably bringing these into sufficiently close proximity to permit autophosphorylation in *trans* and triggering phosphorylation of both the MPL ICD and downstream targets, including signal transducer and activator of transcription (STAT) proteins. Since the TM domain is the site of pathological mutations and the target of small molecule agonists, studies aimed at identifying mechanisms of activation have understandably focused on orientation and rotation of the TM helices. Enticingly, there are some suggestions that differences in TM helical orientation may alter activation status, and this may be responsible for the diversity of signaling output in different cell lineages [40,64]. However, focusing on TM polypeptides in isolation omits key physiological contexts relating to the full-length receptor and associated proteins, and the complex roles played by the plasma membrane.

In contrast, recent studies focusing on the full-length receptor expressed in cell lines suggest that MPL actually exists at the plasma membrane predominantly as monomers that dimerize in response to TPO [11,39,65,66]. Importantly, in cell lines expressing MPL at physiologically relevant levels, TPO-driven receptor dimerization at the plasma membrane monitored by single-molecule imaging followed a bell-shaped dose–response curve, which was perfectly matched by phospho-STAT3 as a readout of downstream signaling, measured using single-cell flow cytometry[11]. This bell-shaped dose-response provides further support for ligand-induced receptor homodimerization via a two-step assembly mechanism (as detailed in Figure 2(a-b) and below).

At physiological receptor densities and in the absence of TPO, MPL and the associated intracellular JAK2 are monomeric and inactive (Figure 2(a-d)). At low concentrations of TPO (below K_1_), most MPL at the cell surface are in the free form, whilst a small number are bound to TPO in a binary (1:1), non-signaling competent complex. As the concentration of TPO rises, the proportion of binary complexes on the cell surface increases, and there is an increased likelihood that these will encounter a second MPL monomer and form a ternary (2:1 MPL:TPO), active complex with the 2-dimensional binding affinity K_2_. Ternary complex formation and downstream signaling reach a maximum when the concentration of TPO equals K_1_. Finally, as the concentration of TPO exceeds K_1_, MPL monomers become saturated with TPO and binary non-signaling complexes again predominate.

Whilst maximum signaling occurs when the concentration of TPO equals K1, the width and amplitude of the bell-shaped curve are functions of K2 and receptor density[66]. An increase in the cell surface receptor concentration will broaden the curve and shift the EC50 to lower ligand concentrations, while a significant decrease in the receptor concentration will reduce the signaling amplitude and shift the EC50 to higher ligand concentration. The impact of receptor density on basal dimerization levels and sensitivity to TPO has important implications for the interpretation of experimental data using cell-line overexpression models (Figure 2(c-d)). In these models, dimerization of MPL and downstream signaling in the absence of TPO, as well as hypersensitivity to TPO may be observed simply as a result of the greater than physiological receptor density.[67]

Although the bridging of two MPL molecules by binding to one molecule of TPO was shown to provide the greatest energetic contribution to receptor dimerization [11], truncated receptors lacking the extracellular domain (and thus incapable of binding TPO) are also able to form dimers [11,65], suggesting that additional interactions, most likely within the TM-JM regions (see next section), contribute to dimer formation. Equally, receptor dimerization by TPO monitored by single-molecule imaging is further increased in the presence of JAK2, consistent with a role for the intracellular JAK2 in stabilizing the receptor dimer at the plasma membrane. Characterization of a series of JAK2 truncations demonstrated that both the FERM-SH2 module and adjoining pseudokinase (PK) domain were required, whilst the C-terminal tyrosine kinase domain was dispensable for receptor dimerization, although required for downstream signaling[11]. The affinities of these additional interfaces, although individually weak, and unable to support a stable dimer for wild-type proteins at low receptor density in the absence of TPO, can be enhanced by mutation to result in cytokine-independent dimerization (Figure 2(c-d) and see next section)[11]. These findings highlight the importance of receptor density and interactions between the receptor, plasma membrane and JAK2.

## Pathological TPO Signaling in Hematological Disorders

Consistent with the critical role of the TPO-MPL signaling axis in HSC maintenance and proliferation, loss-of-function (LOF) and gain-of-function (GOF) mutations in TPO and MPL have been identified as underlying multiple hematological disorders (Figure 1a, Table I) [77,92]. Aberrant signaling arising from GOF mutations results in cellular hyperproliferation leading to hematological malignancies, whilst LOF mutations result in thrombocytopenia and bone marrow failure[77]. A thorough understanding of the regulation of TPO-mediated signaling is important for the diagnosis and treatment of these diseases, whilst the nature of the disease-associated mutations provides insight on regulatory mechanisms. Dysregulation can occur at any of the levels through which the TPO-MPL signaling axis is controlled: from cytokine translation through cytokine and receptor trafficking, secretion, receptor dimerization and downstream signaling.

**Table I.** Mutations in TPO and MPL associated with hematological disorders

Protein	Domain	Mutation	Disease Association	References
TPO	Mpl-binding site 2 (LOF)	R31*, R38C/H/Q	CAMT, aplastic anemia, thrombocytopenia	^[68–70]^
TPO	Mpl-binding (LOF)	R99W, R119C, R157*	CAMT, aplastic anemia	^[22,69–71]^
TPO	C-terminal glycan (LOF)	E204G*fs123, L269P*fs58	Thrombocytopenia	^[22]^
TPO	5ʹ-UTR & splice sites (GOF)	intron 2 (position +2 T > C) intron 3 (G > C, A > G) 1 bp deletion, G516T	Thrombocytosis	^[72–76]^
MPL	CRM1 (GOF)	**T119I, S204F/P** **E230G, Y252H, L265F**	Thrombocytosis	^[77–81]^
MPL	CRM1 (LOF)	K39N, R90*, R102P, P106L	Thrombocytosis (R90*/R102P when heterozygous), ET, PMF	^[49,77,82,83]^
MPL	CRM1 (LOF)	A43*, R90*, R102C/P, F104S, F126*, P136H/L, W154R, S162*, R257C/L, P275T + R102P	CAMT	^[49,82–86]^
MPL	CRM2 (GOF)	V285E, R321W	ET, PMF	^[78]^
MPL	CRM2 (LOF)	W435C	CAMT	^[84]^
MPL	JM-ECD (GOF)	**T487A**	AMKL	^[87]^
MPL	TM (GOF)	**S505N** (germline and somatic) **L498W + H499C, H499G + V501S, H499Y + S505N, V501A + W515L/R, S505N + T487A, S505N + S493C, S505N + V501A/M, S505N + Q516R, S505C + W515L, S505N + V501M + A506V**	ET and PMF	^[65,88–91]^
MPL	JM-ICD (GOF)	**W515A/G/K/L/R/S** (W515R germline and somatic)	ET, PMF	^[77]^
MPL	ICD (GOF)	**Y591D/N**	ET	^[77]^
MPL	ICD (LOF)	R541*, L594W, P635L	CAMT	^[82,84,86]^

Gain of Function (GOF), Loss of Function (LOF), congenital amegakaryocytic thrombocytopenia (CAMT), essential thrombocythemia (ET), primary myelofibrosis (PMF), acute megakaryoblastic leukemia (AMKL). Somatic mutations in **bold**. Residue numbers refer to the amino acid positions in the human TPO and MPL precursor proteins with Uniprot IDs P40225 and P40238, respectively, and thus include the signal peptide (see also Figure 1).

Focusing first on TPO; (a) upregulation of *THPO* translation, and consequent increased serum TPO levels, resulting from point mutations within the 5ʹ-untranslated region (UTR) that remove upstream AUG codons, is a causative factor in hereditary thrombocytosis [72–76]; whilst (b) point mutations (i.e. R99W [22,71]) and truncations (R31*[68], R157*[71]) within the MPL-binding domain, and frameshift mutations within the C-terminal glycan domain[22] result in reduced levels of serum TPO. These latter mutations are causal factors in hereditary thrombocytopenias and bone marrow failure syndromes, and are likely due to mutant protein degradation and/or trafficking defects, potentially caused by protein misfolding. By contrast, TPO R38C and R119C mutants show impaired receptor activation [69,70], possibly as a result of disulfide-mediated TPO dimer formation. Interestingly, R38, R99 and R119 are located on the surface of TPO within the predicted MPL binding site 2 (Figure 1b and c), consistent with a reduced ability to form the ternary signaling complex. The relative paucity of disease-causing mutations identified thus far in *THPO* likely results from the essential nature of this cytokine.

In contrast, multiple LOF and GOF mutations have been mapped throughout the protein-coding sequence of MPL (Figure 1a, Table I). LOF is often associated with lack of receptor at the plasma membrane as a result of premature stop codons, point mutations that impair transcription (P136H), or cause defects in trafficking (K39N, R102P, P106L, W154R, R257C, P635L) (reviewed in Plo *et al*., 2017^77^). Interestingly, R102P has been shown to result in both thrombocytopenia and thrombocytosis, depending on zygosity; in thrombocytosis patients heterozygous for MPL R102P, serum TPO levels were elevated, potentially a result of defective clearance driving hyperproliferation of HSCs expressing WT MPL[83]. Whilst MPL F104S reaches the cell surface, it shows a defect in TPO binding[84], as expected from the location of this residue in the predicted TPO binding site.

Mutations within the MPL TM and JM regions lead to cytokine-independent growth in pre-clinical models [88,93,94] and comprise ~5-10% driver mutations in essential thrombocythemia (ET) and primary myelofibrosis (PMF)[95]. Oncogenic mutations at S505 and W515 have been shown to drive cytokine-independent dimerization via stabilization of the TM helix dimer interface [11,39,63,66]. Saturation mutagenesis of the TM region revealed multiple additional driver and ‘enhancer’ mutations that occur at lower frequency in myeloproliferative neoplasm (MPN) patients [65,89], further highlighting the importance of this region to the active dimer interface.

In addition to mutations in MPL and TPO, multiple disease-associated mutations have been identified in the receptor-associated kinase JAK2 (reviewed in [96]). Here, we will briefly mention only JAK2 V617F, the most prevalent driver mutation in the classical MPNs[97]. JAK2 V617F has recently been shown to bestow cytokine-independence via dimerization of MPL-JAK2 complexes in the absence of TPO through a novel interface within the JAK2 PK domain [11,98,99], thus demonstrating the potential for intracellular drivers of receptor dimerization.

In summary, GOF in MPL, TPO and its associated kinase JAK2 have been shown to result from changes that increase receptor dimerization either via direct stabilization of a dimer interface or via artificially increased levels of circulating TPO resulting from the dysregulation of negative feedback loops. On the other hand, LOF arises as a result of reduction in the levels of circulating TPO or of functional MPL at the plasma membrane (as illustrated in Figure 2(c-d)).

## Perspectives and Concluding Remarks

Although our understanding of the role of the TPO-MPL signaling axis in health and disease has advanced considerably since their genes were cloned in the early 1990s, many questions remain. Not least among these is a more complete molecular understanding of receptor activation and signaling, which will be aided by molecular modeling approaches and the structures of intact signal-transduction complexes. The challenges of recombinant protein production combined with full-length receptor and JAK flexibility have hampered structural studies to date; recent advances in electron cryo-microscopy, alongside solution techniques such as small-angle X-ray scattering [100], look set to deliver more detailed structural information in the near future. Equally, advances in single-molecule imaging have begun to illuminate receptor dynamics on the surface of living cells; however, these techniques have yet to be extended to allow imaging of receptors in their native context on platelets and HSCs. Technical improvements that have increased the sensitivity of biophysical techniques more generally will allow the determination of other key parameters, such as on/off rates and their relationship to downstream signaling, using both live cells and recombinant material. How changes in cytokine-receptor complex half-life and endosomal trafficking fine-tunes cytokine signaling and biological responses requires further investigation.

Together, these new methods will provide a better understanding of the molecular mechanism of MPL activation by TPO and open avenues to the development of novel strategies for the modulation of the pathway. In an important first step, Cui and coworkers have recently discovered and characterized anti-MPL diabodies with differential effects on signaling output[101], which appear to separate the dual roles of MPL signaling; HSC self-renewal and megakaryocyte differentiation. As the development of highly specific synthetic cytokines gathers pace[102] it is tempting to speculate that additional modalities targeting the MPL ECD to alter signaling output could also be achieved.

As our understanding of TPO-MPL signaling evolves, it is now abundantly clear that we cannot consider receptor activation to be a binary “off” or “on”. Instead, it is a tightly regulated continuum, relying on the interplay between cytokine production, membrane receptor density and stability, transmembrane orientation and interactions with JAK2. However, this complexity may provide more diverse therapeutic targets, allowing the precise control of TPO signaling so as to improve HSC self-renewal and better regulate platelet production.
